# A case report of segmental arterial mediolysis in which computed tomography angiography was useful for diagnosis

**DOI:** 10.1007/s12328-013-0433-7

**Published:** 2013-11-06

**Authors:** Haruka Yoshida, Katsuaki Ukai, Mikako Sugimura, Hiromichi Akoshima, Kenji Kimura, Masahiro Iwabuchi, Keiichi Tadokoro, Hiroki Takahashi, Hiroya Rikimaru, Toshihiro Saitoh, Hiroyoshi Suzuki

**Affiliations:** 1 Department of Gastroenterology, Sendai Medical Center, 2-8-8 Miyagino, Sendai, Miyagi 983-8520 Japan; 2 Department of General Medicine, Sendai Medical Center, Sendai, Japan; 3 Department of Radiology, Sendai Medical Center, Sendai, Japan; 4 Department of Surgery, Sendai Medical Center, Sendai, Japan; 5 Department of Pathology and Laboratory Medicine, Sendai Medical Center, Sendai, Japan

**Keywords:** Segmental arterial mediolysis, Maximum-intensity-projection (MIP) images, CT angiography (CTA), Digital subtraction angiography (DSA)

## Abstract

A 48-year-old male presented to our hospital with abdominal pain. Laboratory studies showed no abnormality, the severity of his abdominal pain decreased, and the patient was discharged. Five days later, the patient visited a neighborhood clinic because of fever with a 3-day history of temperatures of approximately 38 °C. The patient was admitted to our hospital 6 days after his initial visit. Laboratory investigation revealed a C-reactive protein level of 18.2 mg/dL. Abdominal computed tomography (CT) showed an 80 × 60 mm hematoma behind the descending colon, but no extravasation was detected. Thin-slice maximum-intensity-projection images from CT angiography (CTA) showed irregular narrowing and intermittent fusiform dilatations of the left colonic artery, suggesting a vascular disease, such as segmental arterial mediolysis (SAM). Digital subtraction angiography showed local irregularity, and ‘beading and narrowing’ of the left colonic artery, similar to the findings on CTA. Left hemicolectomy was electively performed on the twenty-fifth hospital day. Histological findings were consistent with SAM. Thus, CTA was a useful modality for the early diagnosis of SAM.

## Introduction

In 1976, Slavin et al. [1] first described a distinct arterial lesion found in the large abdominal muscular arteries of 3 autopsied patients and called it ‘segmental mediolytic arteritis’. Its chief morphologic characteristic was medial disappearance through an apparent lytic process. It was later revealed that the inflammatory response to this lesion was not uniform and was generally not distributed within the arterial wall. Later, the term ‘segmental arterial mediolysis (SAM)’ was coined [2, 3]. The most common presentations are abdominal pain and hemorrhage in the elderly. Treatment options include conservative care, surgical intervention, and/or endovascular therapy [4, 5]. However, the incidence and mortality of SAM is difficult to accurately estimate because of the rare nature of the disease [6], and optimal therapy for SAM has not been established. Although histopathological examination is the gold standard for diagnosis, patients do not always undergo surgery. Digital subtraction angiography (DSA) is a useful substitute for histopathological diagnosis and can detect specific findings of SAM. DSA features of SAM are arterial dilatations, aneurysms, and occlusions of visceral arteries [7]. Moreover, CT angiography (CTA) can substitute DSA as a non-invasive diagnostic method [8]. We report a case of SAM resulting in an intra-abdominal hematoma in which CTA was useful for the diagnosis.

## Case report

**Fig. 1:**
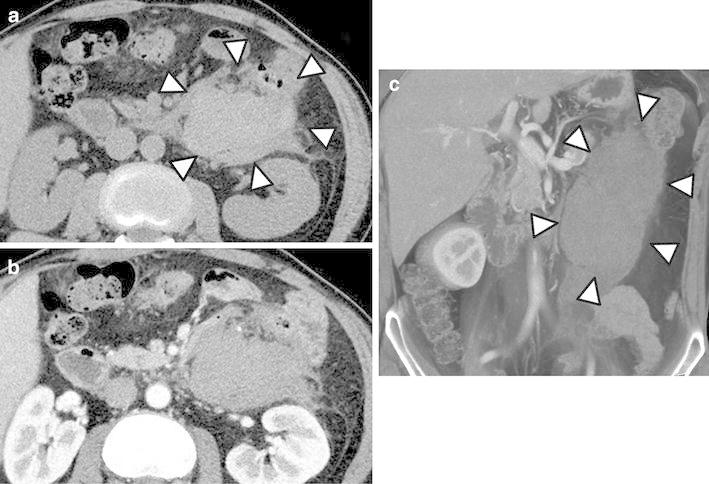
Axial computed tomography (CT) showing an 80 × 60 mm hematoma, surrounded by *arrows* in plain image (**a**), behind the descending colon. **b** The early phase of a contrast-enhanced study shows no stain or extravasation of contrast-enhanced medium. **c** Coronal contrast-enhanced CT shows the hematoma surrounded by *arrows*

**Fig. 2:**
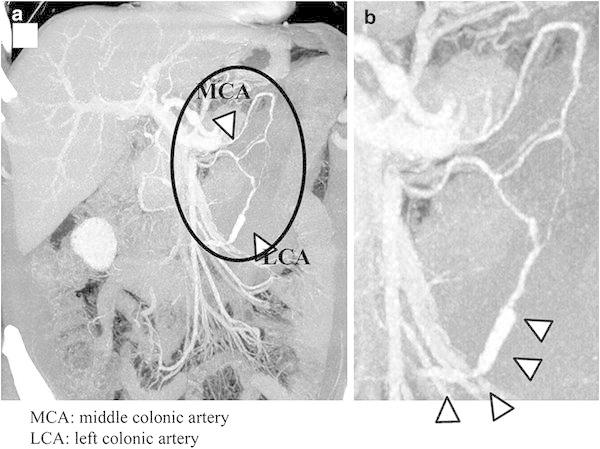
**a** Maximum-intensity-projection (MIP) image from contrast-enhanced coronal CT angiography. **b** Extended figure of part of the circulation in (**a**) demonstrates intermittent arterial dilatation like fusiform aneurysms (*arrow*) in the left colonic artery

**Fig. 3:**
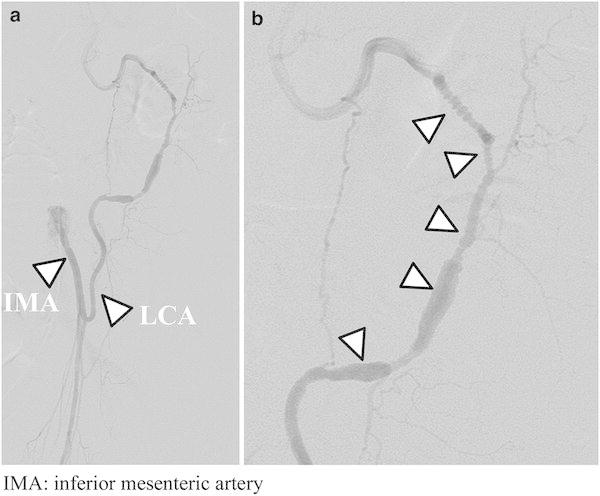
**a** Digital subtraction angiography of the inferior mesenteric artery in the arterial phase. **b** Extended figure of the distal portion of the left colonic artery in (**a**) shows fusiform dilatation and string-of-beads appearance (*arrow*). Extravasation was not detected

**Fig. 4:**
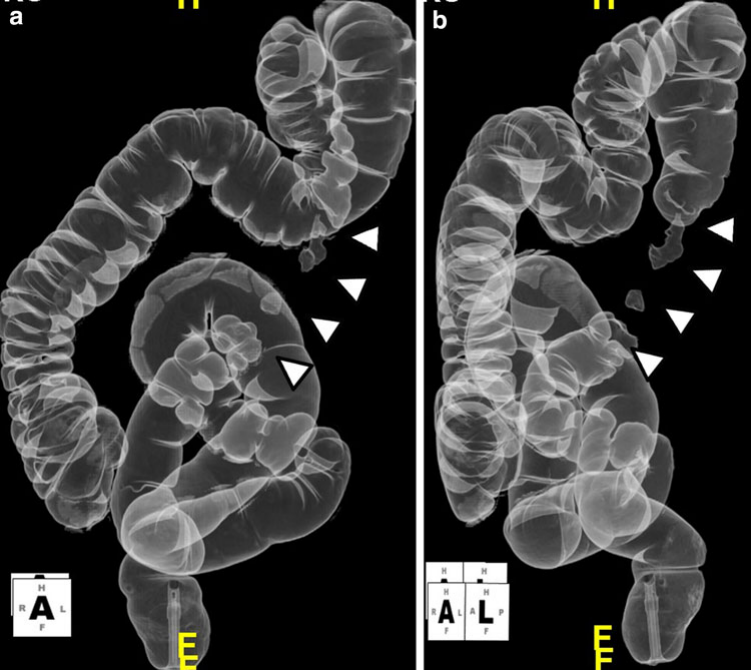
Dorsal position (**a**) and left anterior oblique (**b**) of CT colonography. The descending colon became intermittently blocked by the hematoma (*arrow*)

In April 2012, a 48-year-old male presented to our hospital because of acute left-sided abdominal pain. He had no history of abdominal pain, and he denied a change in bowel habits, loss of appetite, or weight loss. He had no previous medical history. Laboratory studies showed no abnormality, his symptoms resolved without therapy, and he was discharged. Five days later, the patient visited a neighborhood clinic because of fever with a 3-day history of temperatures of approximately 38 °C, and cholecystitis was suspected. The patient was admitted to our hospital 6 days following the initial visit. Laboratory investigation revealed a white blood cell count of 6500/μL, hemoglobin level of 10.5 g/dL, C-reactive protein level of 18.2 mg/dL, and D-dimer level of 12.0 μg/mL (Table 1). Computed tomography (CT) on admission showed no evidence of cholecystitis. However, an 80 × 60 mm hematoma was detected behind the descending colon (Fig. 1). Thin-slice maximum-intensity-projection (MIP) images from CTA showed fusiform dilatations and irregular narrowing of the left colonic artery, suggesting the involvement of vascular disease including SAM (Fig. 2). During hospitalization, the patient’s symptoms were relieved with conservative therapy, and atherosclerosis, fibromuscular dysplasia, vasculitis, and connective tissue disease were ruled out. On the fifth hospitalization day, DSA showed no extravasation of contrast medium, but detected fusiform dilatation and a ‘string-of-beads appearance’ of the left colonic artery (Fig. 3). We therefore diagnosed the patient with SAM. The lesion was detected from the relatively proximal side of the left colonic artery. We decided against performing transarterial embolization (TAE), which could induce massive ischemia in the descending colon. CT colonography showed that the descending colon became intermittently blocked owing to the hematoma (Fig. 4). Colonoscopy detected no ischemic change in the mucosa, but the colonoscope and barium did not pass through the obstructive lesion. On the nineteenth hospitalization day, although CT showed that the size of the hematoma was reduced (52 × 35 mm), CTA showed that the arterial irregularity was still present. The patient was considered a candidate for surgery because of the risk of ileus and rerupture. On the twenty-fifth hospital day, a left hemicolectomy was performed, and the patient was discharged on the fifty-second hospital day.

**Table 1:** Laboratory examination on admission

WBC	6500/μL	TP	6.9 g/dl
RBC	337 × 10^4^/μl	Alb	3.8 g/dl
Hb	10.5 g/dL	T-Bil	1.1 mg/dl
Ht	31.5 %	AST	28 IU/l
Plt	31.3 × 10^4^/μl	ALT	44 IU/l
		LDH	170 IU/l
PT	100 %	ALP	448 IU/l
PT-INR	1.00	Na	139 mEq/l
Fib	880 mg/dl	K	4.7 mEq/l
FDP	23 μg/ml	Cl	99 mEq/l
D-dimer	12.0 μg/ml	BUN	16 mg/dl
		Cr	0.69 mg/dl
		CRP	18.2 mg/dl

**Fig. 5:**
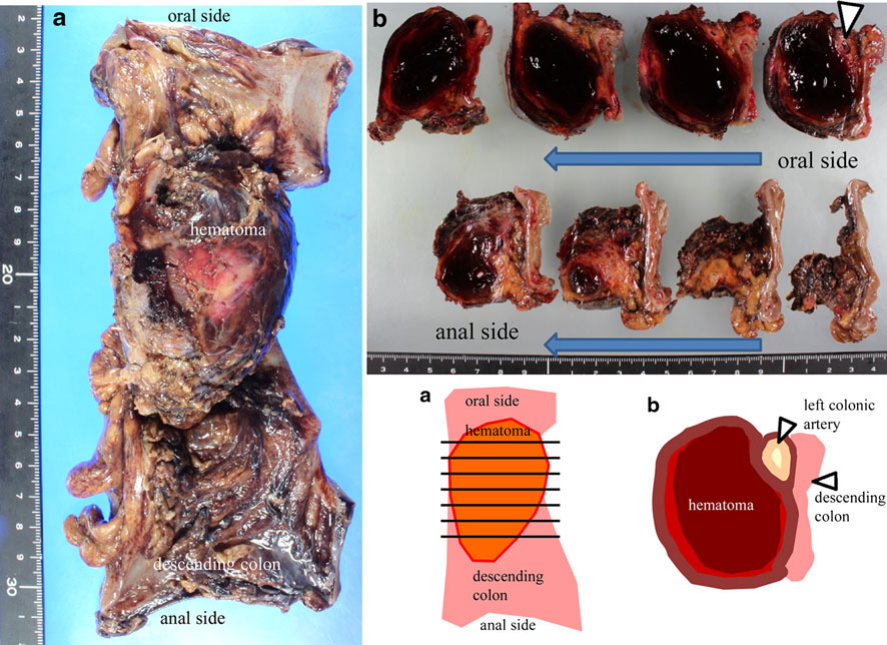
**a** Resected specimen revealed a 70 × 45 × 40 mm hematoma in the side of the serosa of the descending colon. **b** Cut surfaces of the serial section of the hematoma: the left colonic artery was distended and was present in the mid-portion of the hematoma (*arrow*)

**Fig. 6:**
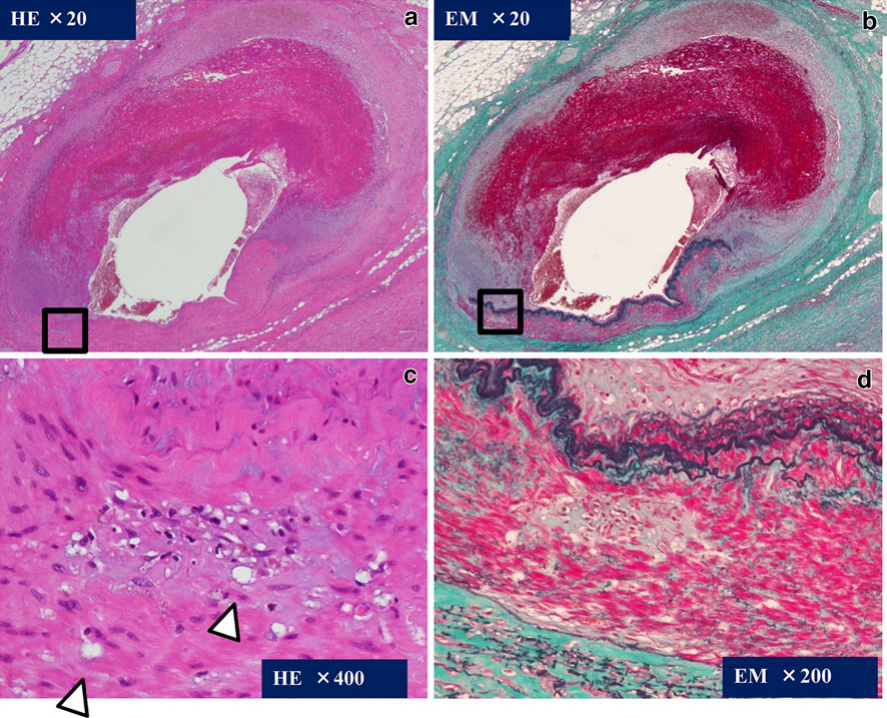
Histopathological examination of the resected left colonic artery. **a** Hematoxylin and eosin staining (×20) and **b** Elastica-Masson staining (×20) show that the wall of the artery is partially dissected with intramural hemorrhage. **c** Higher magnification (×400) of a *square part* of (**a**) shows vacuolar degeneration of smooth muscle cells in the tunica media (*arrow*). **d** Higher magnification (×200) of the *square part* of (**b**) shows irregular-shaped degeneration of the tunica media with focal fibrosis

Macroscopically, a tumorous hematoma was protruding from the serosal surface of the colonic wall. Examinations by serial sectioning of the hematoma identified the presence of a medium-sized artery with dilation of the lumen continuous to the proximal part of the hematoma (Fig. 5). Histological examination of the abnormal artery and hematoma disclosed segmental thinning of the internal elastic lamina and insular degeneration and vacuolization of smooth muscle cells of the media with patchy fibrosis. Intramural hemorrhages in the dissecting media of the artery were also observed. These findings were considered to be the underlying pathological processes leading to aneurysm formation (Fig. 6), and were consistent with SAM and with our clinical diagnosis. Thus, CTA was able to detect the typical findings of SAM as detected by DSA.

## Discussion

Michael et al. [8] described SAM as a rare non-arteriosclerotic, non-inflammatory vascular disease of unknown origin that involves the visceral arteries and has no predilection for bifurcations. SAM primarily affects the outer layer of the media, leading to vacuolar degeneration of smooth muscle cells. The disruption of vacuoles and concomitant loss of their fluid contents ultimately results in disruption of the media, intramural hemorrhage, and periadventitial fibrin, thrombi, or granulation tissue, which can lead to saccular aneurysms, dissecting aneurysms, or thrombosis. SAM most commonly involves the large abdominal aortic branches, considered to be medium-sized vessels. A review of 52 cases by Inada et al. [9] revealed the middle colic artery to be the most frequently involved vessel (38.4 %), followed by the gastroepiploic arteries (19.2 %) and gastric arteries (17.3 %). Involvement of the left colic artery was seen in only 1 case (1.9 %), indicating that ours is a relatively rare case.

The differential diagnosis of SAM includes atherosclerosis, fibromuscular dysplasia, infection (e.g., mycotic aneurysm and endocarditis), connective tissue disease (e.g., Behçet’s disease and polyarteritis nodosa), neurofibromatosis, and inherited defects in vessel wall structural proteins (e.g., type IV Ehlers–Danlos syndrome and Marfan’s syndrome) [10, 11]. The clinical findings and symptoms in patients with vasculitis are non-specific and quite variable.

Although histopathology is the gold standard for definitive diagnosis, vascular tissue is available only in patients undergoing surgery [8]. Thus, Uchiyama et al. [12] proposed clinical diagnostic criteria for SAM, which include (1) middle-aged and elderly patients, (2) no pre-existing disease such as inflammatory disease and arteriosclerotic disease, (3) sudden onset with intra-abdominal hemorrhage, and (4) angiographically detected ‘string-of-beads appearance’ in the abdominal visceral arteries. Our patient met all these criteria, and we were able to make the diagnosis of SAM prior to the histological examination.

However, angiography cannot necessarily be performed in all cases either owing to its invasive nature or because a hospital lacks the facility. We could find only 1 report that considers the role of CTA compared with DSA in the diagnosis of SAM, as below. Michael et al. [8] compared CTA results with those of DSA in 4 cases, and both CTA and DSA identified the characteristic findings of SAM in all cases. They concluded that CTA provided sufficient evidence of SAM. In our case, MIP images from CTA identified the characteristic findings of SAM, which are similar to those identified by DSA, and were extremely useful in making the diagnosis.

In the MIP method, viewing rays are traced from the expected position of the operator through the object to the display screen, and only the relative maximum value detected along each ray path is retained by the computer. This method tends to display bone and contrast material-filled structures preferentially [13]. In the diagnosis of SAM, MIP images of CTA findings are similar to those of DSA, indicating that this technique could replace DSA. The diagnosis of SAM could be much easier to make using the MIP method for CTA, which may in turn lead to the clarification of epidemiological data on SAM.

Several treatments of SAM have been reported in each distinct clinical presentation [5]. Surgical treatments include laparotomy with urgent segmental resection of the affected bowel, ligation, or excision of an aneurysm [14], and surgical reconstruction with autologous grafts [15]. Moreover, some cases were treated by TAE with coils or *N*-butyl cyanoacrylate [11, 12], or balloon angioplasty [16]. A standard therapy for SAM has not been established, so the best treatment for each case must be selected in consideration of vital signs, CTA or DSA findings, etc.

Ishizaki et al. [4] reported a case of aneurysm rupture in the abdominal visceral artery caused by SAM, which was successfully managed with conservative therapy. However, there is no consensus on a definitive therapy because the long-term natural history of SAM is yet to be defined. Moreover, Oya et al. [17] reported a case of aneurysm re-rupture caused by SAM, and another author reports that mortality in the acute phase of this disease is close to 50 % [6]. Therefore, we believed that our patient required appropriate treatment. In our case, DSA did not detect extravasation, so transcatheter arterial embolization was not performed. In addition, the patient did not require emergency surgery because his vital signs were stable and his symptoms were immediately relieved. However, we considered that he might develop obstructive ileus by hematoma and re-rupture of the abnormal vessels. As a result of consultation with the surgeon and sufficient informed consent from the patient, he underwent an elective left hemicolectomy.

Although a definitive therapy has not been established, SAM occurs mostly with acute intra-abdominal hemorrhage and can be critical in some cases, making early diagnosis extremely important. The MIP method of CTA is thought to be useful as a non-invasive and commonly usable technique for early diagnosis.
